# A Short Digital Food Frequency Questionnaire (DIGIKOST-FFQ) Assessing Dietary Intake and Other Lifestyle Factors Among Norwegians: Qualitative Evaluation With Focus Group Interviews and Usability Testing

**DOI:** 10.2196/35933

**Published:** 2022-11-08

**Authors:** Hege Berg Henriksen, Markus Dines Knudsen, Monica Hauger Carlsen, Anette Hjartåker, Rune Blomhoff

**Affiliations:** 1 Department of Nutrition Institute of Basic Medical Sciences University of Oslo Oslo Norway; 2 Section for Colorectal Cancer Screening Cancer Registry of Norway Oslo Norway; 3 Department of Transplantation Medicine Division of Surgery, Inflammatory Diseases and Transplantation Oslo University Hospital, Rikshospitalet Oslo Norway; 4 Department of Epidemiology Harvard TH Chan School of Public Health Boston, MA United States; 5 Division of Cancer Medicine Oslo University Hospital Oslo Norway

**Keywords:** digital assessment tool, assessment tool, food frequency questionnaire, food, diet, nutrition, questionnaire, focus group, interview, usability, physical activity, lifestyle factor, dietary intake, digital health, chronic disease, chronic condition, health promotion, cancer, survivor, usability, thematic analysis, research tool, measurement tool

## Abstract

**Background:**

In-person dietary counseling and interventions have shown promising results in changing habits toward healthier lifestyles, but they are costly to implement in large populations. Developing digital tools to assess individual dietary intake and lifestyle with integrated personalized feedback systems may help overcome this challenge. We developed a short digital food frequency questionnaire, known as the DIGIKOST-FFQ, to assess diet and other lifestyle factors based on the Norwegian Food-Based Dietary Guidelines. The DIGIKOST-FFQ includes a personalized feedback system, the DIGIKOST report, that benchmarks diet and lifestyle habits. We used qualitative focus group interviews and usability tests to test the feasibility and usability of the DIGIKOST application.

**Objective:**

We aimed to explore attitudes, perceptions, and challenges in completing the DIGIKOST-FFQ. We also investigated perceptions and understanding of the personalized feedback in the DIGIKOST report and the technical flow and usability of the DIGIKOST-FFQ and the DIGIKOST report.

**Methods:**

Healthy individuals and cancer survivors were invited to participate in the focus group interviews. The transcripts were analyzed using thematic analysis. Another group of healthy individuals completed the usability testing, which was administered individually by a moderator and 2 observers. The results were analyzed based on predefined assignments and discussion with the participants about the interpretation of the DIGIKOST report and technical flow of the DIGIKOST-FFQ.

**Results:**

A total of 20 individuals participated in the focus group interviews, divided into 3 groups of healthy individuals and 3 groups of cancer survivors. Each group consisted of 3 to 4 individuals. Five main themes were investigated: (1) completion time (on average 19.1, SD 8.3, minutes, an acceptable duration), (2) layout (participants reported the DIGIKOST-FFQ was easy to navigate and had clear questions but presented challenges in reporting dietary intake, sedentary time, and physical activity in the last year), (3) questions (the introductory questions on habitual intake worked well), (4) pictures (the pictures were very helpful, but some portion sizes were difficult to differentiate and adding weight in grams would have been helpful), and (5) motivation (users were motivated to obtain personalized feedback). Four individuals participated in the usability testing. The results showed that the users could seamlessly log in, give consent, fill in the DIGIKOST-FFQ, and receive, print, and read the DIGIKOST report. However, parts of the report were perceived as difficult to interpret.

**Conclusions:**

The DIGIKOST-FFQ was overall well received by participants, who found it feasible to use; however, some adjustments with regard to reporting dietary intake and lifestyle habits were suggested. The DIGIKOST report with personalized feedback was the main motivation to complete the questionnaire. The results from the usability testing revealed a need for adjustments and updates to make the report easier to read.

## Introduction

The Norwegian Food-Based Dietary Guidelines (Norwegian FBDG), published by the Norwegian health authorities, aim to reduce the risk of lifestyle-related chronic diseases and promote overall health in the general Norwegian population [1]. There is a lack of, and therefore a need for, easily accessible dietary and lifestyle assessment tools with a low respondent burden, (eg, by being feasible, quick to complete, motivational to use, and able to collect accurate, precise lifestyle data) [2-5]. Motivation is a crucial factor when recruiting people to undertake the challenge of filling in questionnaires. Personal feedback has been used in other studies to motivate study participants [6-8]. Thus, a digital diet and lifestyle questionnaire tool that automatically gives personalized feedback, including health and diet-related advice, would theoretically increase completion in future studies.

Digital applications assessing diet and lifestyle behaviors for use in epidemiological and clinical studies are emerging [2,6,8-12]. For instance, a web-based, semiquantitative food frequency questionnaire (FFQ) assessing habitual diet over the last year was found to be feasible for use among healthy adults living in Norway [13] and served as a valuable tool to be used in epidemiological studies. However, this web FFQ was solely developed to collect data on dietary intake, without any digital application for individual feedback reports. Forster et al [3] developed a dietary feedback system based on dietary intake, Food4Me FFQ, which was found to be well accepted by participants and feasible for use [8]. Moreover, the MyFood decision-support system was developed to assess symptoms and dietary intake among hospitalized patients at risk of malnutrition and to generate reports on personalized nutritional treatment for use by nurses or other health care professionals [9,14,15]. Another digital application for clinical use is eCHANGE, developed as a personalized digital intervention aiming at providing self-management support for long-term weight maintenance [12].

To the best of our knowledge, no digital application has been developed to assess dietary intake according to the Norwegian FBDG with integrated personalized feedback reports. We developed the DIGIKOST-FFQ, a short, digital, semiquantitative food and lifestyle frequency questionnaire, designed to assess adherence to the Norwegian FBDG. The DIGIKOST-FFQ is applicable in a number of settings where information on diet and lifestyle is needed. Based on a respondent’s answers to the DIGIKOST-FFQ, a report, known as the DIGIKOST report, is automatically generated and immediately made available to the respondent after completion. It gives individual feedback on the respondent’s adherence to the Norwegian FBDG and on other lifestyle factors, as well as advice on how to fulfill the recommendations.

The process of creating new digital dietary and lifestyle assessment tools involves several developmental stages, from defining the different constructs in the questionnaires to making it feasible for use. In addition, new research tools must be evaluated to explore their validity and reproducibility [16]. To evaluate digital tools that assess diet or lifestyle, qualitative methods, such as focus group interviews, are increasingly being used, constituting mobile health (mHealth) [16-18]. Focus groups are particularly useful for exploring people’s knowledge and experiences, and can be used to examine not only what people think, but also how they think and why they think the way they do [19,20]. Results from focus group interviews are used in the further development of the tools in question.

During a focus group interview, the participants are invited to share their views, comments, and perspectives, phrased in their own words and in synergy with the other participants in the group. A moderator ensures that the structure and framework of the interview follow the focus group interview guide [19,20].

Usability testing is also a key component in the development of digital applications [21,22] and is critical in the development and improvement of the design, function, and understanding of the tool being developed. It is performed by real users trying to accomplish typical goals and tasks in a test version of the digital tool under controlled conditions, allowing researchers and the development team to observe and take notes [21,22]. Results from these observations are then used in the development of the tool.

As part of the development of the DIGIKOST-FFQ, we performed several focus group interviews with healthy individuals and cancer survivors, because both of these groups are expected to be important study populations in future research studies using the DIGIKOST-FFQ. Furthermore, an independent group of healthy individuals was invited to the usability testing of both the DIGIKOST-FFQ and the DIGIKOST report. In the current paper, we present the results from the focus group interviews and the usability testing of the DIGIKOST-FFQ and the DIGIKOST report.

## Methods

### The DIGIKOST-FFQ

The DIGIKOST-FFQ is derived from a paper-based, validated, short, semiquantitative food frequency questionnaire called the NORDIET-FFQ [23,24], which was designed to measure adherence to the Norwegian FBDG. The first draft of the DIGIKOST-FFQ that underwent evaluation in the current study consisted of 80 questions on diet; 5 on physical activity, time being sedentary, and sleeping; 10 on tobacco use; and 9 on demographic data. In addition, the questionnaire included introductory questions about the usual intake of specific food groups. If a participant indicated no intake of any food item in a food group, the participant was redirected to the next food group. Moreover, when reporting no intake for a specific food item, the associated question on amount disappeared, due to an automatic function in the questionnaire, and the participant was redirected to the next food group.

### The DIGIKOST Report

The DIGIKOST report presents adherence to the Norwegian FBDG in different ways (Multimedia Appendix 1). First, dietary intake and physical activity are estimated from the DIGIKOST-FFQ and presented in a table that compares the results with the Norwegian FBDG. Next, the same components are presented graphically, with columns presenting the degree of adherence to the Norwegian FBDG, measured as a percentage with traffic-light coloring. In the next section, adherence to the recommendations is presented as a health index consisting of 5 lifestyle components: diet, weight status (BMI), physical activity, smoking, and intake of alcohol. Each component is equally weighted. The degree of adherence is divided into a 3-level scoring system for diet, weight status, and physical activity, ranging from no adherence (0 points) to intermediate adherence (0.5 points) and full adherence (1 point). For alcohol and tobacco use, the degree of adherence is binary (0 or 1). The total health index ranges from 0 to 5 points. The participant’s achievements in the health index score are presented and compared with the maximum score of the index. In addition, benchmarking of recorded individual diet and other lifestyle factors against the Norwegian FBDG is presented, along with individual advice on how to fulfill the recommendations. At the end of the report, the Healthy Eating Plate is presented (Multimedia Appendix 1). The Healthy Eating Plate is a commonly used model that focuses on diet quality. The plate features 3 individual sections: one-third should consist of vegetables, one-third of whole grains and starchy vegetables, and one-third of fish, meat, or legumes [25].

### Subjects

Both healthy adult individuals and cancer survivors were recruited to the focus group interviews, while only healthy individuals were recruited to the usability testing. Healthy individuals were recruited using Facebook announcements, with separate recruitment processes for focus group interviews and the usability testing. Cancer survivors were recruited from an ongoing randomized controlled trial, the CRC-NORDIET study, and from the Norwegian Cancer Society user group panel [26,27]. The recruitment period for both studies was from April to June 2020. The focus group interviews and the usability testing were all conducted remotely by video meetings on Zoom due to the COVID-19 pandemic in 2020 and 2021. Group sizes of 5 to 10 and 4 to 5 individuals are recommended for focus group interviews and usability testing, respectively [28,29].

### Focus Group Interviews

A moderator and 2 researchers from the Department of Nutrition at the University of Oslo (UiO) led the focus group interviews. Recording of the interviews was done with both Zoom and the Dictaphone app for smartphones, which sent all data directly to a secure server, the Services for Sensitive Data (“Tjenester for sensitive data,” abbreviated “TSD” in Norwegian), at the University Center for Information Technology (USIT) at UiO [28]. In addition, the moderator and the assistants recorded feedback with written notes. All recorded data were safely stored at TSD. The focus group interviews were conducted according to the interview guide for focus groups that is included as part of the DIGIKOST-FFQ (Multimedia Appendix 2). The DIGIKOST reports were not ready for use at the time of the focus group interviews; therefore, the focus group interviews only tested the DIGIKOST FFQ and not the DIGIKOST reports. However, the focus group participants were asked whether a personal report on their individual lifestyles would motivate them to complete the DIGIKOST-FFQ.

All transcripts of the recorded focus group interviews were made with f4transkript software (version 6.2.5 Pro; Dr. Dresing & Pehl GmbH) [30]. The transcripts were analyzed by taking notes and coming to an overall understanding of the basic responses (ie, themes) and creating codes and constructs according to the DIGIKOST-FFQ interview guide for focus groups (Multimedia Appendix 2). The results were stratified into groups comprising healthy individuals and cancer survivors. Analysis of the transcripts was done manually and independently by 2 researchers, HBH and MDK, to identify the main themes and constructs in the responses. Afterwards, a thorough evaluation of the results was performed collaboratively by the same researchers. The most frequent responses were noted, as well as the single response evaluated as most improving the feasibility of the DIGIKOST-FFQ; these were included in the revised version.

### Usability Testing of the DIGIKOST Report

**Figure 1:**
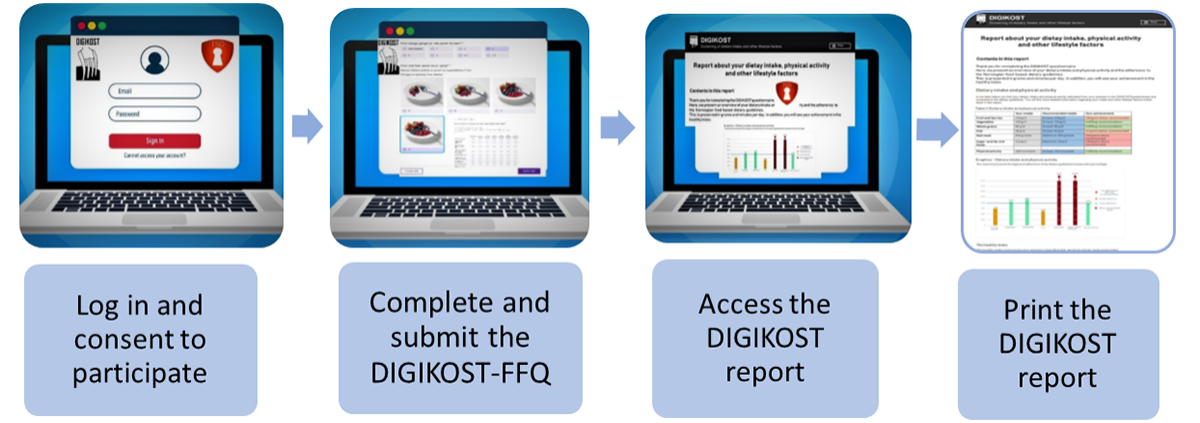
Technical flow of usability testing.

The usability testing was completed individually and conducted on Zoom with each participant. At this time, the DIGIKOST report was ready for using and testing. A moderator from USIT at UiO led the usability testing, with 2 observers from the Department of Nutrition at UiO taking notes on how the participants completed the planned tasks. The purpose was to test the technical flow of the DIGIKOST-FFQ, from consent and completion of the DIGIKOST-FFQ to opening and printing the individual DIGIKOST reports (Figure 1).

Furthermore, after printing the DIGIKOST report, the participants shared their understanding and views of the report (Multimedia Appendix 1) by answering questions about (1) their understanding of the graphs in the report, (2) their comprehension of the specific recommendations for diet and health improvements, (3) their conception of the table, and (4) their conception of the health index included in the report. The usability testing was performed according to a standard protocol developed by USIT [31,32].

### Ethics

The current study was carried out in accordance with the Helsinki Declaration; informed consent was obtained from all participants. The Norwegian Centre for Research Data approved the focus group protocol, the usability testing, and the informed consent (277679). All participants signed the informed consent form before completing the DIGIKOST-FFQ, focus group interviews, and usability testing. After accepting the study invitation, the participants were asked to fill out the DIGIKOST-FFQ before the focus group interviews. For the usability testing, the participants did not have access to the DIGIKOST-FFQ or the DIGIKOST report before attending the testing.

## Results

### Focus Group Interviews

A total of 20 adults, including 11 women and 9 men, participated in 6 focus group interviews, with 3 to 4 participants in each group. Each interview lasted for 1 to 1.5 hours. The focus group discussions had a natural flow, but were guided by the motivator using the interview guide. Five main themes and subthemes were identified through analysis of the transcripts: (1) the time it took to complete the questionnaire, (2) the layout of the questionnaire, (3) the questions in the questionnaire, (4) the pictures of portion sizes in the questionnaire, and (5) motivations for the participant to fill out the questionnaire (Table 1).

In general, the responses from the 2 groups of participants were similar and addressed the same themes and topics. However, cancer survivors had a harder time reporting physical activity and time being sedentary than healthy individuals. Healthy individuals asked for more questions about plant-based food and a third gender option.

**Table 1:** Summary of responses from the focus groups on main themes obtained from a discussion that was based on the interview guide included in the DIGIKOST-FFQ. Results are stratified by group.

Main themes		Responses from healthy individuals (7 females, 3 males)	Responses from cancer survivors (4 females, 6 males)
**Completion time**			
	Completion time (minutes), average (SD, range)	17.2 (4.5, 10-25)	21.1 (11.1, 15-45)
	Main comments about completion time	“Got tired at the end”; “the questionnaire cannot be longer”	“I have no belief in completing in 15 minutes”
**Layout**			
	What themes worked well	Differentiation of portion sizes, ease of navigation, clarity of questions, reporting physical activity	Having to answer every question, automatic calculation of bread slices
	What themes did not work well	Questions on money spent on snuff; reporting intakes in the previous year, over several seasons, or of foods you eat less than once a week when the answer is equal to never; and about duration of residence in Norway when you have answered that you were born in Norway	Questions about money spent last year or season on snuff, eggs, or jam; sedentary time; physical activity; and about duration of residence in Norway when you have answered that you were born in Norway. Pictures of glasses filled with alcoholic beverages would have been helpful
**Questions**			
	Yes/no as introduction to a food group	Worked well, could have been implemented for all thematic question groups	Worked well
	Missing questions/options	Questions were missing on plant-based cold cuts, alternative milk products (eg, soy, oat, or rice), and potatoes; there were only 2 categories for answers on marital status; add student as a separate category from education; Norway should be at the top of the pull-down choice list of countries; there were no answer options for a third category for gender or separate categories for full-time and part-time work	Questions were missing on legumes, eggs, and potatoes; Norway should be at the top of the pull-down choice list, “transport” was not included in the answer options for sedentariness; no option for power naps
**Pictures**			
	Portion size	Informative text should have been added to the pictures and some portions, such as for A, B, C, and D, which were difficult to tell apart	Informative text should have been added to the pictures and some portions, such as for A, B, C, and D, which were difficult to tell apart
	Were the pictures helpful or could they have been text only?	Pictures that included weight measurements in grams would have been helpful; references to the pictures would improve the information, as would more text before new food items and pictures	Pictures of better quality would help reporting quantitative intake; weight measurements in grams should have been included as references in the pictures; more text should have been included before new food items and pictures
**Motivation**		The individual reports were motivational	The individual reports were motivational

#### Completion Time

All participants completed the DIGIKOST-FFQ within a scheduled time of about 15 to 20 minutes. However, many respondents stressed that the questionnaire should not take more time than this to fill out:

I see that I got tired at the end...

Healthy female participant; June 22, 2020

I noticed that in the end it became more skim reading.

Healthy male individual; June 22, 2020

However, the participants also stated that the digital format was easier than a paper questionnaire:

It is much simpler compared to the whole paper mill where you have to sit for hours to complete.

Female cancer survivor; May 8, 2020

#### Layout

Both groups agreed that the DIGIKOST-FFQ was easy to navigate and the questions were easy to understand:

I think it was easy to navigate and there was a good progression and so, you were not surprised by any of the questions. The questions appeared clear and concise and easy to understand.

Healthy male individual; June 22, 2020

...and then the question and portion sizes are simple and straight forward. Period.

Male cancer survivor; May 8, 2020

The advantage of including different kinds of questions in the questionnaire to prevent it from becoming boring and tiring toward the end was also emphasized:

I believe in general that it is good if you change the way you ask the questions, so you do keep yourself awake and not just “click” your way down.

Healthy female individual; March 23, 2020

A questionnaire to be completed with a lot of fun...

Healthy male individual; June 23, 2020

We observed 2 challenges with the questionnaire: reporting seasonal variation in dietary intake and registering time being sedentary:

We are living in a society with big variation according to season. Especially in the north where the access to fruit and vegetables is determined by season.

Healthy female individual; June 12, 2020

I believe that it is very season dependent what we eat now and what we eat later. In the winter you eat paprika, whereas in the summer you eat strawberries, which has just started now.

Healthy male individual; June 23, 2020

I found it (sedentary time) seriously complicated to answer, I didn’t have a chance.

Female cancer survivor; June 22, 2020

I agree (sedentary time), I was very... I have no idea, however then I tried to picture an ordinary day, but I think there were a huge difference between what I pictured and what I noted for an ordinary day, to say it that way.

Healthy male individual; June 22, 2020

#### Questions

The introductory questions were well accepted by the participants:

It worked very well.

Female and male healthy individuals; June 12, 2020

I think... for my part it fitted very well, you know... I can say “no” to things that I for sure never eat, and when I say “yes” to something, I pretty much found what I eat. However, I am probably more average when it comes to diet [laughter].

Male cancer survivor; May 12, 2020

It worked very well.

Female cancer survivor; April 14, 2020

I think it was okay to answer yes or no, I had no problems with that.

Healthy female individual; June 22, 2020

There were a few alternative answer options that the participants felt were missing from the questionnaire, such as categories for gender:

I noticed that there were only two variables [for gender].

Healthy male individual; June 22, 2020

Yes, in modern society you probably should have a third alternative for gender. And I, working at a health clinic for rare diseases, know that this exists.

Healthy female individual; June 12, 2020

Moreover, the cancer survivors emphasized the importance of being able to include power naps and transportation time in the definition of sedentary time. It was suggested that a function for summing the different levels of physical activity (eg, time being sedentary, sleeping, or engaging in physical activity) throughout a 24-hour period should be included to increase the feasibility of reporting those questions correctly. The cancer survivors also felt that questions were missing about intake of legumes, potatoes, and eggs. Reporting intakes of berries used in homemade jam was also challenging for some, because they were unsure whether to report homemade jam as jam in the questions about spreads or in the questions about berries.

#### Pictures

**Figure 2:**
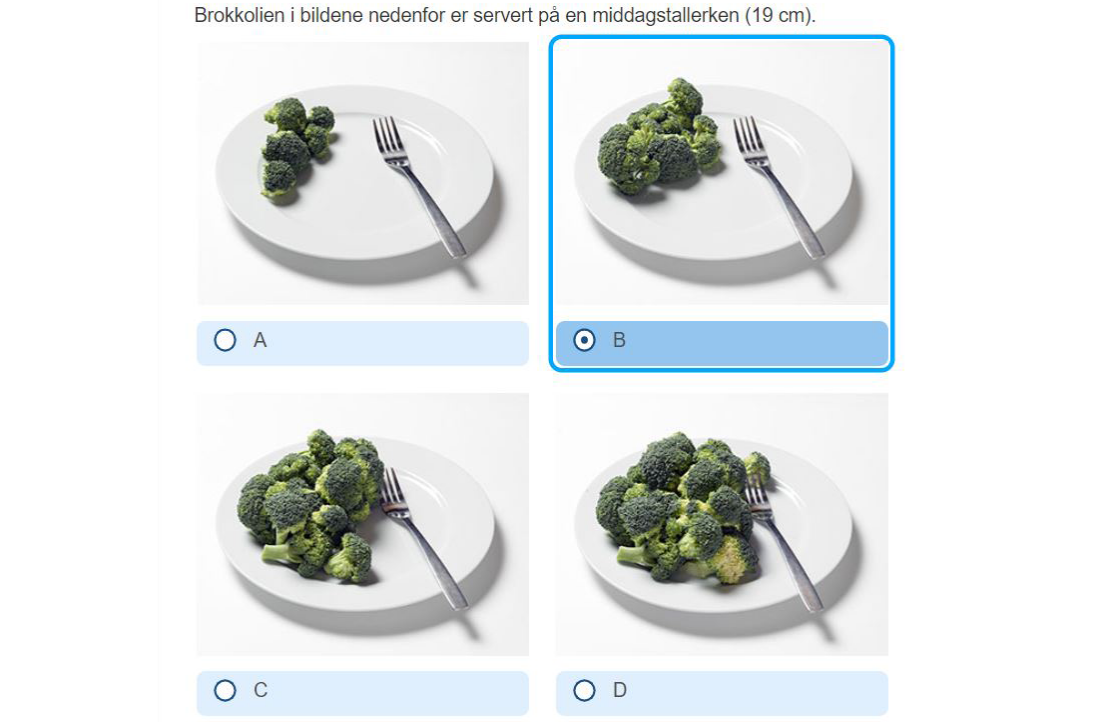
Example of a question from the DIGIKOST-FFQ regarding intake of broccoli, with 4 illustrations of portion sizes.

There was a request for more text and the addition of grams to each image with portion sizes to increase the accuracy of the reporting. In particular, for some participants, it was difficult to tell some portion illustrations apart, especially portion sizes A and B and portion sizes C and D, as illustrated in Figure 2.

Some of the participant responses were as follows:

Now we are closing in on where I really missed grams for references.

Male cancer survivor; May 8, 2020

Beautiful pictures, particularly the berries.

Female cancer survivor; April 14, 2020

#### Motivation

A report about adherence to the Norwegian FBDG, along with a trending curve showing the change in intake over time (for studies where the participants would fill in the DIGIKOST-FFQ repeatedly over time), would have motivated participants in both groups to complete the DIGIKOST-FFQ:

I would like to find out more about my diet and lifestyle out of curiosity.

Healthy female individual; June 23, 2020

I think that some kind of report about your lifestyle, would be great fun.

Healthy female individual; June 22, 2020

Yes, it is obvious, it is fun to get feedback. See how you are placed... What kind of actions should I take.

Healthy female individual; June 22, 2020

It would be nice with feedback and compare your current to how you did in previous rounds... see if you have improved or worsened, preferably in tables or figures.

Male cancer survivor; May 12, 2020

### Usability Testing

A total of 4 of 5 invited individuals, including 2 women and 2 men, participated in the usability testing. All attendees showed good technical skills. There was wide variation in their educational backgrounds, ages, and residential locations in Norway (data not shown). It took approximately 1 hour to conduct the usability testing for each attendee. All participants completed the 4 test tasks (Figure 1). However, due to technical issues with the ID portal, 1 of the participants could not test the first 3 steps in the technical flow. Therefore, that participant completed the questionnaire without the log-in function. Moreover, due to a technical issue, the DIGIKOST reports were emailed to the participants after completion of the DIGIKOST-FFQ, instead of appearing automatically online. This technical issue has now been resolved.

#### Technical Flow

The 3 participants who completed the technical flow testing (ie, the first 3 steps of the test) of the DIGIKOST-FFQ performed well, but 1 of the participants had minor challenges completing and submitting the DIGIKOST-FFQ (Table 2).

**Table 2:** Summary of participant performance in the usability and technical flow testing, based on the usability protocol.

Technical flow steps	Participant 1	Participant 2	Participant 3	Participant 4
1. Log in and consent to participate	Passed	Passed	Passed	Did not pass due to technical issues
2. Complete and submit the DIGIKOST-FFQ	Passed	Passed, but with minor issues	Passed	Did not pass due to technical issues
3. Access the personal DIGIKOST report via email	Passed	Passed	Passed	Did not pass due to technical issues
4. Print the DIGIKOST report	Passed	Passed	Passed	Passed

#### Interpretation of the DIGIKOST Report

The participants were, in general, positive about the DIGIKOST report (Multimedia Appendix 1). The coloring used to indicate adherence to the recommendations was well accepted, because it illustrated the degree of adherence in a clear manner. Three of the participants found the first table informative, whereas 1 preferred to look at the graphic with adherence shown as a percentage rather than read the table (because there was too much text). The report also included a graph showing adherence to the recommendations as a percentage. When a respondent had a higher intake of a food group than the minimum recommended amount, such as if they ate more than 5 fruits or vegetables a day, they would get a score of above 100%. All participants understood the graphic showing adherence to the recommendations as a percentage. However, 1 participant pointed out that the concept of percentages >100% could be challenging to understand.

The aim of the health index included in the report appeared to be unclear and difficult to understand for most participants. One participant asked whether the aim was to increase your BMI to achieve a full score by increasing dietary intake. Another participant found it difficult to see the difference between the 2 indices (ie, BMI and health index). Some found it difficult to understand the total sum score of 5 points when each component in the score reached only a maximum of 1 point. The participants suggested either improving the presentation of the health index by making it simpler or removing it from the report entirely. The participants liked the use of traffic-light coloring; however, it was pointed out that the colors used could be challenging for individuals who are color blind.

All participants reacted positively to the immediate individual response with advice on how to improve adherence to the recommendations, and 1 pointed out that it was very helpful. Another participant suggested reorganizing the responses by presenting the advice on improving adherence to the recommendations first and presenting the recommendations that were fulfilled second.

## Discussion

### Principal Findings

The DIGIKOST-FFQ, with the DIGIKOST report, is the first short digital FFQ and personal report that has been benchmarked against the Norwegian FBDG. The findings from both the focus group interviews and the usability testing showed that the DIGIKOST-FFQ and the DIGIKOST report were overall well accepted and easy to use. However, the study also revealed challenges for users and a need for some improvements. Particularly for the DIGIKOST-FFQ, there were challenges related to reporting seasonal variation in dietary intake over the last year, reporting physical activity, and differentiating images illustrating different portion sizes. FFQs rely on memory and a participant’s conceptualization of portion size and frequency of intake, and these are frequently mentioned as challenges in the literature [33-35]. Seasonal variation in dietary intake is another well-known challenge when using FFQs that collect data from several seasons or ask about intake of foods that are specifically seasonal [33-35]. For the DIGIKOST report, interpretation of the graphics and percentages in the histograms was the main challenge.

To understand the willingness to respond and the motivation to participate in surveys and thereby improve survey effectiveness, de Leeuw et al [36] used an attitude scale to measure survey attitudes. Results from this study revealed 3 dimensions representing important contributors to participation in surveys: survey enjoyment (this reflects the individual perception of surveys as a positive experience), survey value (ie, salience, relevance, and usefulness), and survey burden (ie, if the survey is perceived as a burden on the individual, it has a negative influence on motivation and participation). All participants in the current study acknowledged the value of the DIGIKOST-FFQ and the importance of monitoring diet and lifestyle factors in the prevention of disease and improvement of quality of life. They also experienced survey enjoyment through the pleasant design of the questionnaire. Pictures and other visual aids (eg, reference objects, household measures, and food packaging) have been shown to be preferable and beneficial when assessing portion sizes and helpful in improving the accuracy of food quantification [5,37,38]. This is in accord with the current study, where pictures of portions were perceived as helpful, although adding more information, such as amounts in grams or household measures, was suggested as a way to increase the usability even more.

All participants agreed that the questionnaire should be short and take no more than 15 to 20 minutes to complete to reduce the survey burden and maintain enjoyment and motivation to participate. Previous studies have found that the use of digital dietary assessment tools is perceived as more fun, more motivational to use, and preferable to paper-based dietary assessment tools [2,4,39,40]. This was also supported by the participants in the current study.

Overall, the results from the focus group interviews showed that there were no large differences in the feedback from the healthy individuals and the cancer survivors, indicating that completion of the DIGIKOST-FFQ was equally feasible for both groups. However, some differences in their feedback should be pointed out. The cancer survivors found it most challenging to report daily activities and intake of traditional foods, whereas the challenges identified by the healthy individuals were related more to social status and the lack of questions about novel food products available on the market today. We assume that the differences in the feedback from healthy individuals and cancer survivors might be due to age; however, we do not have information on the age of the participants.

The results from the usability testing showed that the technical flow of the questionnaire was good. Most participants found the DIGIKOST reports easy to understand, and all enjoyed the individual advice and recommendations presented in the text at the end of the report. However, some preferred a more visual presentation of the results, such as percentage adherence, rather than the textual information in the table. We speculate that it would be difficult to please all individual preferences on how to visualize the results, and that the solution might therefore be to include both tables and graphics in the report to suit both preferences. The interpretation of the health index varied to a great extent, and most participants found it difficult to understand. Difficulties in perceiving health risk factors presented as percentages or other statistical terms have been documented in patients with low numeracy, whereas interactive graphics may be more easily perceived [41-43]. Thus, when communicating health risk factors, it is important to be aware that different formats generate different risk perceptions among patients with different levels of numeracy [42].

### Strengths and Limitations

DIGIKOST-FFQ is accessible from multiple electronic devices, such as personal computers, phones, and tablets, allowing for high flexibility in future use of the tool, minimal respondent burden, and potentially reduced selection bias. Further advantages are a low demand for personnel and economic resources and easy implementation in research settings, including both observational and interventional studies; the literature has reported similar advantages for previous digital questionnaires [2]. Moreover, the questions in the digital platform are easy to change and adapt to follow future updates in dietary and lifestyle guidelines. A strength of our study is the inclusion of both healthy individuals and cancer survivors in the focus group interviews. Moreover, we included new participants in the usability testing to make sure it was the first time the participants tested the DIGIKOST-FFQ and DIGIKOST reports so that we could obtain their first-impression feedback of its usability. Another strength is that the participants had a good variety of backgrounds (in gender, work and education, and location in Norway).

A limitation of the current study could be that the focus group interviews were carried out by video call on Zoom due to the COVID-19 pandemic, which could have affected the group dynamics and the ability of the participants to freely discuss their ideas. Some participants found it difficult to participate on Zoom due to technical issues and low-quality sound or video, and we speculate that the study population may have been biased toward people with access to high-quality digital equipment and greater technological skills. Nevertheless, the video-call format made it possible to include individuals living across Norway.

### Future Improvements in DIGIKOST-FFQ and the DIGIKOST Report

The focus group interviews contributed valuable knowledge about users’ challenges and led to suggestions for improvements. For instance, in the future, we will include more text about each food item and present food quantities by weight or volume, in addition to pictures representing different portion sizes. Moreover, as most of the participants found it challenging to report intake and activities over the previous year due to seasonal variations, the time frame of reporting will be revised and reduced to the previous 2 months.

Several aspects of the DIGIKOST reports were challenging to understand for the participants. Therefore, we will remove the graphics showing achievements as percentages, the health index, and the Healthy Eating Plate recommendations. Moreover, we will add more lifestyle factors to the “individual advice for you” section, such as alcohol intake, smoking, physical activity, and body weight.

### Conclusions

The DIGIKOST-FFQ and the DIGIKOST report were well received by the participants, who found it easy to log in to and navigate the system and understand the questions. The completion time was acceptable. Changes in the questionnaire and report to address difficulties in recalling dietary intake over the previous year and due to seasonal variation will be implemented. Also, text with additional information on weight or volume will be added to the portion-size pictures. All participants found it motivational to receive personalized feedback reports with dietary advice. The usability testing showed that the log-in system worked well, but that some adjustments were needed to the reports in order to make the personalized feedback more understandable.

## Appendix

Multimedia Appendix 1 DIGIKOST report, first draft.

Multimedia Appendix 2 Interview guide for the focus groups on DIGIKOST-FFQ.

## Abbreviations

CRC-NORDIET study: The Norwegian dietary guidelines and colorectal cancer survival study: a food-based multicenter randomized controlled study
FBDG: Food-Based Dietary Guidelines
FFQ: food frequency questionnaire
ID: Identification data
TSD: Tjenester for sensitive data (Services for Sensitive Data)
UiO: University of Oslo
USIT: University Center for Information Technology
