# Supplementary material for: A Short Digital Food Frequency Questionnaire (DIGIKOST-FFQ) Assessing Dietary Intake and Other Lifestyle Factors Among Norwegians: Qualitative Evaluation With Focus Group Interviews and Usability Testing
Source: JMIR Form Res. 2022 Nov 8;6(11):e35933. doi: 10.2196/35933 (PMC9682459; doi:10.2196/35933)
Supplement: Multimedia Appendix 1 [file formative_v6i11e35933_app1.pdf]

DIGIKOST
Screening of dietary intake and other lifestyle factors

Print

Report about your dietay intake, physical activity and other lifestyle factors

Contents in this report

Thank you for completing the DIGIKOST questionnaire.
Here, we present an overview of your dietary intake and physical activity and the adherence to the Norwegian food based dietary guidelines.
This is presented in grams and minutes per day . In addition, you will see your achievement in the healthy index.

Dietary intake and physical activity

In the table below you find your dietary intake and physical activity estimated from your answers in the DIGIKOSTquestionnaire and compared to the dietary guidelines. You will find more detailed information regarding your intake and other lifestyle factors further down in this report.

Table 1: Dietary intake and physical activity

	Your intake	Recommended intake	Your achievement
**Fruit and berries**	150 g/d	At least 250 g/d	100 gram below recommended
**Vegetables**	260 g/d	At least 250 g/d	Fulfilling recommendation
**Whole grains**	90 g/d	At least 90 g/d	Fulfilling recommendation
**Fish**	28 g/d	At least 43 g/d	15 grams below recommended
**Red meat**	856 g/week	Maximum 500 g/week	356 grams above recommended
**Sugar- and fat rich foods**	210 g/d	Maximum 20 g/d	190 grams above recommended
**Physical activity**	180 min/week	At least 150 min/week	Fulfilling recommendation

Graphics - Dietary intake and physical activity

The columns presents the degree of adherence of the dietary guidelines measured in percentage.

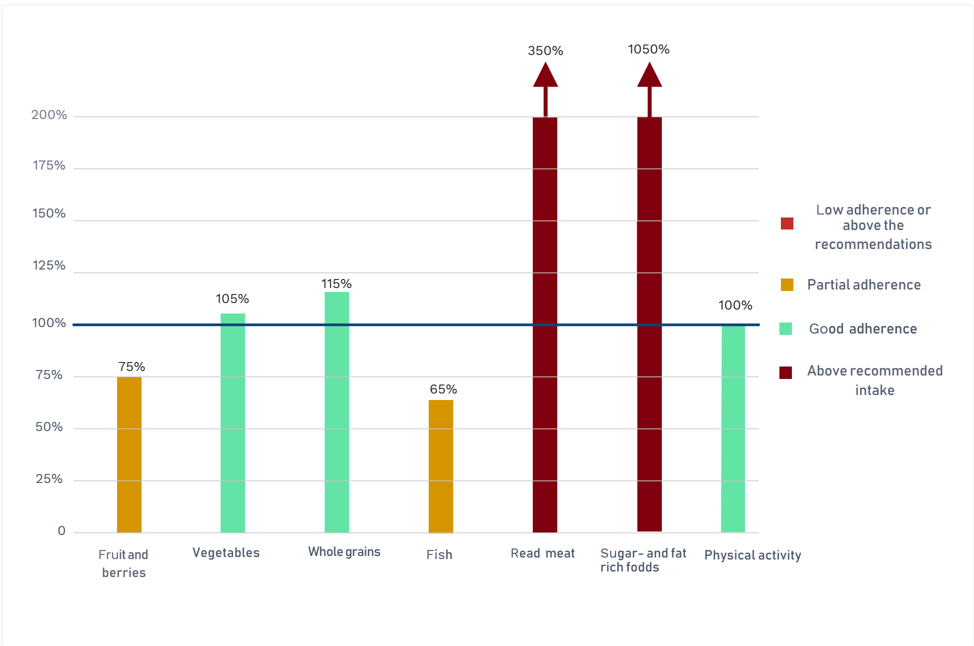

The healthy index

The healthy index summaries your answers regarding diet, physical activity, body mass index (BMI) and usage of tobacco and alcohol. The figure presents your achievements in the index for each component in the index as well as the total achievement. The maximum score is 5 points.

- Diet indicates your total adherence to the dietary guidelines
- It is recommended to be in at least 150 minutes physical activity in moderate intensity per week.
- It is recommended to be normal weighed with a BMI between 18 and 25.
- It is not recommended to use tobacco or alcohol

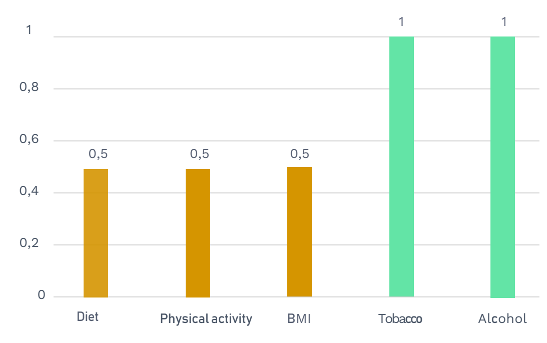

Healthy index score
3.5 out of 5 points

Your achievement in the healthy index score is 3.5 out of 5 points. Your diet and physical activity is partly good, but with room for improvement, your BMI is above normal.

You achieved full adherence to the recommendations regarding not using tobacco and alcohol and this is very good!

Individual advices for you

Based on your reported dietary intake and other lifestyle factors, we have made some specific advices in how to fulfil the recommendations. You can by only small adjustments increase your benefits in health.

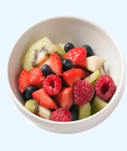

You eat some fruit, but less than recommended

You eat 150 grams of fruit per day. It is great that you eat some fruit, however, if you increase the intake with one banana and an apple per day you will reach the recommended daily intake.

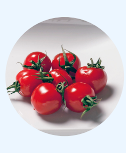

You eat the daily recommended amounts of vegetables

You eat 260 grams of vegetables per day. Keep on, it benefits your health

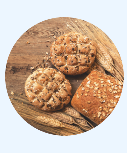

You eat the daily recommended amounts of whole grains

You eat 90 grams of whole grains per day. Keep on, it benefits your health

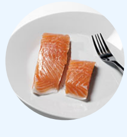

You eat some fish, but less than recommended

You eat 28 grams of fish per day. It is good that you eat some fish, but if you increase the intake by a slice of salmon or cod (120 grams) for dinner and use fish as spread to your lunch, you will reach the recommended intake

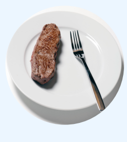

You eat more red meat than recommended

You eat 856 grams of red meat per week. We recommend you to limit intakes of red meat to a maximum of 500 grams per week, or red meat for dinner 2-3 times a week.

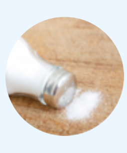

Reduce intakes of sugar- and fat rich foods

Your intake of sugar- and fat rich foods is higher than recommended. Try to replace with foods containing less or no sugar and fat.

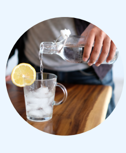

Reduce intakes of sugar-rich drinks

Your intake of sugar-rich drinks is higher than recommended. Try to replace with drinks containing less or no sugar.

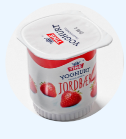

You eat recommended amounts of low-fat dairy products

You eat 20 grams of low-fat dairy products per day. Keep on, it its good for your health.

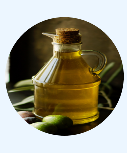

You follow the recommendation of using oils and soft margarines

You follow the recommendations of oils and soft margarines as the primarily choice in cooking. Keep on, it is good for your health.

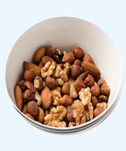

You do not follow the recommendation of eating nuts

You do not eat nuts. If you increase your nut intake by at least 20 grams per day, you will achieve what is recommended.

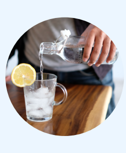

You drink less water than recommended

Your intakes of water is less than other drinks per day. Water is good for your health, and if you increase the intake with one to two glasses per day, you will reach the recommendations.

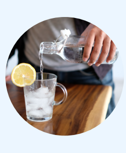

You follow the recommendation of no alcohol intake

You do not have intakes of alcoholic drinks. Keep on, it is good for your health.

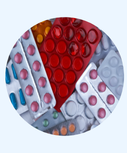

You follow the recommendation of dietary supplements

You do not have any intake of dietary supplements. Unless you are adviced by your physician to have an intake, you should keep on not having an intake.

Plate model

A healthy dinner contains a lot of vegetables
Imagine you to split your plate into three equal pieces.

- 1/3 vegetables
- 1/3 potatoes, whole grain rice and pasta
- 1/3 fish, meat or legumes

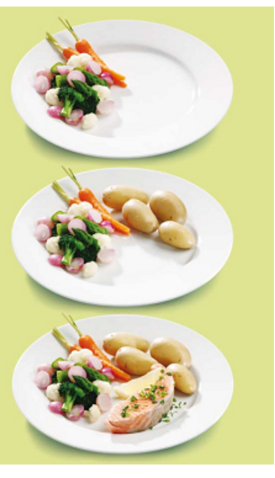

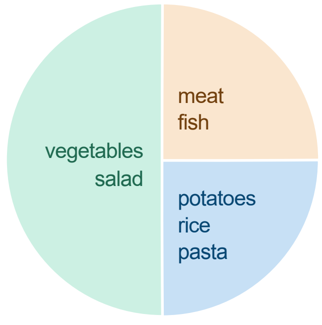

Do you want to reduce your body weight? Change your plate model to:

- 1/2 vegetables or salad
- 1/4 potatoes,rice or pasta
- 1/4 meat or fish
