# Supplementary material for: A Short Digital Food Frequency Questionnaire (DIGIKOST-FFQ) Assessing Dietary Intake and Other Lifestyle Factors Among Norwegians: Qualitative Evaluation With Focus Group Interviews and Usability Testing
Source: JMIR Form Res. 2022 Nov 8;6(11):e35933. doi: 10.2196/35933 (PMC9682459; doi:10.2196/35933)
Supplement: Multimedia Appendix 2 [file formative_v6i11e35933_app2.docx]

## Multimedia Appendix 2. Interview guide for the focus groups on DIGIKOST-FFQ.

## 1. Introduction and start up 10 min

- Present the moderator and assistant
- Today’s team: evaluation of DIGIKOST-FFQ
- Explain about the focus group interview and what is going to be used for, development of the questionnaire that they have completed
- Anonymity
- Recording of the interview
- The focus groups interview will last for approximately 2 hours
- No right or wrong answers
- The participants do not have to agree with each other, but they do have to respect each other’s opinions
- Explain the role of the moderator and that this is a conversation between the participants and not between the moderato and the participants.
- Before we start please turn of you mobile phone or put it away so it do not disturb
- We are on a first name basis
- Presentation of the participants around the table, it is voluntary for each participant on how much information they want to give.

## 2. Layout 15-20 min

- What worked well with the questionnaire?
- What did not work so well with the questionnaire?
- How did it work answering yes/no to sub groups of food?
- How was the text, was it of any help? E.g. the text for carrots

Ask the participants to elaborate, if necessary take a round around the table

## 3. The questions 15-20 min

- How were the questions formulated?
- Where there any of the questions that was specifically difficult to understand?
- Any question/teams you missed when you think about lifestyle?
- If you should have formulated the questions, how would you have put them?

Ask the participants to elaborate, if necessary take a round around the table

## 4. Portion sizes 15-20 min

- How were the portion sizes compared to what you usually eats?
- How where the variation on the portion sizes?

Ask the participants to elaborate, if necessary take a round around the table

## 5. The pictures 15-20 min

- Where the pictures of any help?
- Could it had been only text ?
- How were the quality of the pictures?
- How were the size of the pictures?
- Were there any questiosn that should have had pictures?
- How did the demonstration pictures work (e.g. onion = chopped onions, meat)?

Ask the participants to elaborate, if necessary take a round around the table

## 6. Motivation for completing the DIGIKOST-FFQ 15-20 min

- What kind of motivation would you need to complete this questionnaire?
- How did you experience the time used?
- How would you prefer to be contacted to answer this questionnaire?
- Would you consider completing the DIGIKOST-FFQ on other platforms that a computer?

Ask the participants to elaborate, if necessary take a round around the table

## 7. Ending 5 min

- If you were to invite some of your friends to answer this questionnaire, what would you say?
- Imagine that you were to design the questionnaire. How would you do it, what would you do differently and what would you have focused on?
- Any last suggestions for improving the questionnaire?
- The moderator ask the assistant if there is any further questions?
- Thank you for participating
